# Emerging patterns of genome organization in Notopteridae species (Teleostei, Osteoglossiformes) as revealed by Zoo-FISH and Comparative Genomic Hybridization (CGH)

**DOI:** 10.1038/s41598-019-38617-4

**Published:** 2019-02-04

**Authors:** Felipe Faix Barby, Luiz Antônio Carlos Bertollo, Ezequiel Aguiar de Oliveira, Cassia Fernanda Yano, Terumi Hatanaka, Petr Ráb, Alexandr Sember, Tariq Ezaz, Roberto Ferreira Artoni, Thomas Liehr, Ahmed B. H. Al-Rikabi, Vladimir Trifonov, Edivaldo H. C. de Oliveira, Wagner Franco Molina, Oladele Ilesanmi Jegede, Alongklod Tanomtong, Marcelo de Bello Cioffi

**Affiliations:** 1 Departamento de Genética e Evolução, Universidade Federal de São Carlos (UFSCar), Rodovia Washington Luiz Km. 235, C.P. 676, São Carlos, SP 13565-905 Brazil; 2 Laboratory of Fish Genetics, Institute of Animal Physiology and Genetics, Czech Academy of Sciences, Rumburská 89, Liběchov, 277 21 Czech Republic; 3 Institute for Applied Ecology, University of Canberra, Canberra, ACT 2617 Australia; 4 Departamento de Biologia Estrutural, Molecular e Genética, Universidade Estadual de Ponta Grossa, Ponta Grossa, PR 84030-900 Brazil; 5 Institute of Human Genetics, University Hospital Jena, 07747 Jena, Germany; 6 Molecular and Cellular Biology, Russian Academy of Sciences, Novosibirsk, Russia; 7 Laboratório de Cultura de Tecidos e Citogenética, SAMAM, Instituto Evandro Chagas, Belém, Brazil; 8 Department of Cellular Biology and Genetics, Biosciences Center, Federal University of Rio Grande do Norte, Natal, Brazil; 9 Department of Fisheries and Aquaculture, Adamawa State University, P.M.B. 25, Mubi, Adamawa State Nigeria; 10 Toxic Substances in Livestock and Aquatic Animals Research Group, KhonKaen University, Muang, KhonKaen, 40002 Thailand

## Abstract

Notopteridae (Teleostei, Osteoglossiformes) represents an old fish lineage with ten currently recognized species distributed in African and Southeastern Asian rivers. Their karyotype structures and diploid numbers remained conserved over long evolutionary periods, since African and Asian lineages diverged approximately 120 Mya. However, a significant genetic diversity was already identified for these species using molecular data. Thus, why the evolutionary relationships within Notopteridae are so diverse at the genomic level but so conserved in terms of their karyotypes? In an attempt to develop a more comprehensive picture of the karyotype and genome evolution in Notopteridae, we performed comparative genomic hybridization (CGH) and cross-species (Zoo-FISH) whole chromosome painting experiments to explore chromosome-scale intergenomic divergence among seven notopterid species, collected in different African and Southeast Asian river basins. CGH demonstrated an advanced stage of sequence divergence among the species and Zoo-FISH experiments showed diffuse and limited homology on inter-generic level, showing a temporal reduction of evolutionarily conserved syntenic regions. The sharing of a conserved chromosomal region revealed by Zoo-FISH in these species provides perspectives that several other homologous syntenic regions have remained conserved among their genomes despite long temporal isolation. In summary, Notopteridae is an interesting model for tracking the chromosome evolution as it is (i) ancestral vertebrate group with Gondwanan distribution and (ii) an example of animal group exhibiting karyotype stasis. The present study brings new insights into degree of genome divergence vs. conservation at chromosomal and sub-chromosomal level in representative sampling of this group.

## Introduction

The monophyletic fish order Osteoglossiformes represents an ancient teleost group with a geographic distribution restricted to the freshwater river basins^1–3^. This ancestral teleost lineage retained primitive anatomical features (e.g., the toothed tongue bones)^4^ and, considering their very ancient origin (200 Mya), their current distribution pattern could reflect the vicariance events occurring after the Gondwana’s break-up^5,6^. While some osteoglossiform families (Gymnarchidae, Mormyridae, and Pantodontidae) are restricted to Africa, other ones (Osteoglossidae, Notopteridae, and Arapaimidae) exhibit a patchy distribution, with species endemic to different continents^7^.

Their wide geographic distribution and basal position in the teleost phylogeny, qualifies this group as an excellent model for systematic, genomic, cytogenetic and evolutionary studies. However, only few cytogenetic and genomic studies have been undertaken so far, covering a limited number of osteoglossiform species [reviewed in^8–13^]. This lack of data is probably due to the worldwide distribution of the whole group and, on the other hand, the endemic status of majority of its species, thus hindering integrative studies that allow a globalized view of its evolutionary process.

Presently, the Notopteridae family includes four genera (*Chitala*, *Notopterus*, *Xenomystus*, and *Papyrocranus*) with ten recognized species^14^. While *Chitala* and *Notopterus* are endemic to Southeastern Asia, *Papyrocranus* and *Xenomystus* exhibit wide distribution throughout the African freshwater habitats^15^. Representatives of this family are known as “featherfishes”^16^ and are restricted to freshwaters, except for a single species - *N. notopterus*, which can be occasionally found in brackish waters^15^. *Chitala* is the most speciose genus encompassing at least six recognized species, with several of them being commercially important for both aquaculture and the aquarium trade. While *X. nigri* and *N. notopterus* are not likely monotypic^6^, the remaining notopterid genus *Papyrocranus* has two species (*P. afer* and *P. congoensis*)^15^. Phylogenetic studies support the monophyletic status of Notopteridae, placing them within the suborder Notopteroidei, together with Gymnarchidae and Mormyridae, whose representatives are found exclusively in African rivers^7,17^.

An integrative study of Barby *et al*.^9^ combining molecular-cytogenetic data with DArTseq (Diversity Array Technology Sequencing) SNP markers was recently performed in seven notopterid species. In brief, this study showed that, from the cytogenetic standpoint, five species displayed karyotypes composed of 2n = 42 exclusively acrocentric chromosomes, while *Papyrocranus afer* and *Chitala lopis* showed 2n = 50 and 2n = 38, respectively. For the last species, interstitial telomeric sites (ITSs) were observed in the first and third chromosomal pairs, thereby providing strong evidence for the involvement of fusion events as the mechanism beyond the reduction of 2n. Nonetheless, SNP analyses demonstrated a great differentiation at genome level between congeneric species and distinguished two major Notopteridae clades based on the genomic and chromosomal features – i.e. the African and the South Asian species groups. The major finding of that study was thus the contrasting scenario showing, on one hand, a significant genetic diversity observed among these species while, on the other hand, the retention of the conservative karyotype macrostructure which is clearly apparent. The question then arises: why the evolutionary relationships within Notopteridae are so diverse at the genomic level but so conserved in terms of their karyotypes? Which evolutionary drivers have facilitated these contrasting scenarios between modes of karyotype and genome evolution?

In recent years, an increasing drive towards application of advanced molecular cytogenetic tools in studies conducted in fishes is apparent. Namely, comparative genomic hybridization (CGH) and chromosome painting techniques (WCP) have gained a prominence in solving various issues in the field of comparative cytogenetics in fishes, allowing access to more fine-scale insights into a number of evolutionary issues. Put succinctly, especially over the last decade, both technologies have been used in fishes for investigation of (1) the genome divergence between closely related species^18–24^; (2) the evolution and gross-scale molecular composition of sex chromosomes^21,22,24–26^ and (3) the origin of B chromosomes^27–29^. Besides that, both CGH and related method genomic *in situ* hybridization (GISH) were successfully employed also in studies aiming in parental genome identification among asexual polyploid or homoploid fish hybrids^13,20,30^ or in studies focused on the uniparental chromosome elimination^31,32^.

In this study, we performed CGH and Zoo-FISH experiments to explore intergenomic divergence at the chromosomal level among seven out of 10 extant species of the Notopteridae family; the sampling resembles the one previously analyzed by Barby *et al*.^9^ with different cytogenetic and molecular methods. Indeed, our recent results provided new insights into the karyotype differentiation of this fish group, with both methodologies proving to be valuable for gaining a better understanding of genomic and chromosomal differentiation, highlighting relationships and uncovering chromosome homologies and differences among studied species.

## Results

### Whole chromosome painting of XN-1 probe

**Figure 1:**
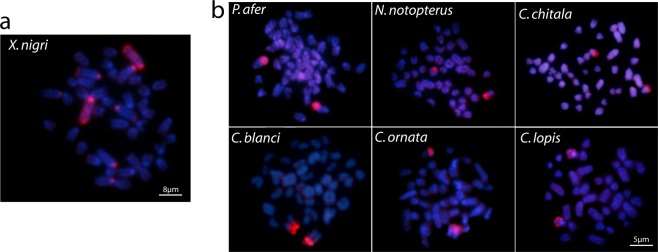
Zoo-FISH results after the use of Xn-1 probe. (**a**) XN-1 probe applied in the metaphase plate of *X. nigri* and (**b**) The XN-1 probe hybridization in the other six notopterid species.

As a control experiment, we first applied the XN-1 probe (prepared from the first chromosome pair of the *X. nigri* complement) back against the *X. nigri* metaphase plates and the first chromosome pair was completely painted, as expected; with prominent hybridization signals in both terminal regions. Additionally, faint hybridization blocks in the centromeric region of three other chromosomal pairs were also observed **(**Fig. 1a**)**.

The hybridization of XN-1 probe in the remaining set of six Notopteridae species showed a bright hybridization signals in the centromeric regions of a medium-sized acrocentric pair in all of them. However, despite the impossibility to accurately identify this chromosome pair, it seems to represent orthologous chromosomes taking into account their similar size and morphology **(**Fig. 1b).

### Comparative genomic hybridization (CGH)

**Figure 2:**
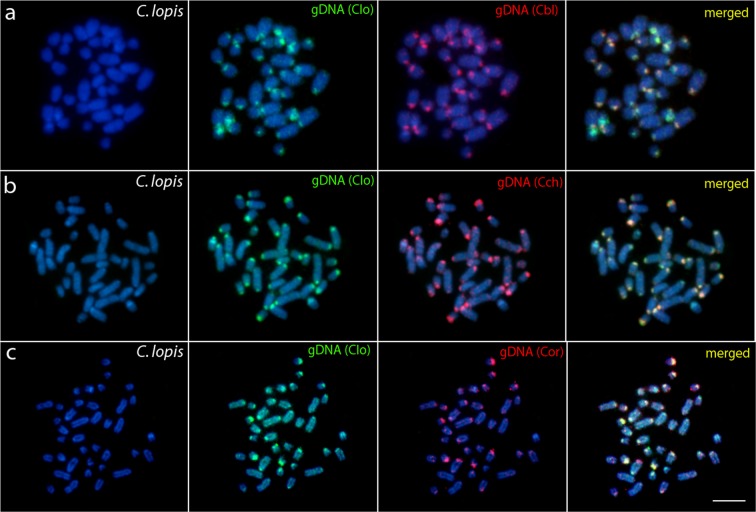
Comparative genomic hybridization (CGH) using the gDNA of *C. blanci* (**a**), *C. chitala* (**b**) *and C. ornata* (**c**) against chromosomal background of *C. lopis*. First column: DAPI images (blue); Second column: hybridization pattern with *C. lopis* (Clo) gDNA probe; Third column: hybridization patterns with *C. blanci* (Cbl) gDNA, *C. chitala* (Cch) gDNA and *C. ornata* (Cor) gDNA. Fourth column: merged images of both genomic probes and DAPI staining. The common genomic regions are depicted in yellow. Bar = 5 μm.

**Figure 3:**
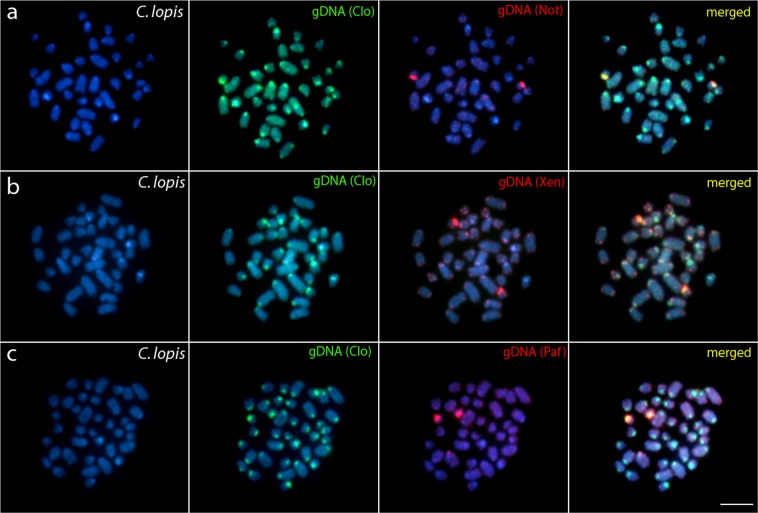
Comparative genomic hybridization (CGH) using the gDNA of *N. notopterus* (**a**), *X. nigri* (**b**) *and P. afer* (**c**) against chromosomal background of *C. lopis*. First column: DAPI images (blue); Second column: hybridization pattern with *C. lopis* (Clo) gDNA probe; Third column: hybridization patterns with *N. notopterus* (Not) gDNA, *X. nigri* (Xen) gDNA and *P. afer* (Paf) gDNA. Fourth column: merged images of both genomic probes and DAPI staining. The common genomic regions are depicted in yellow. Bar = 5 μm.

**Figure 4:**
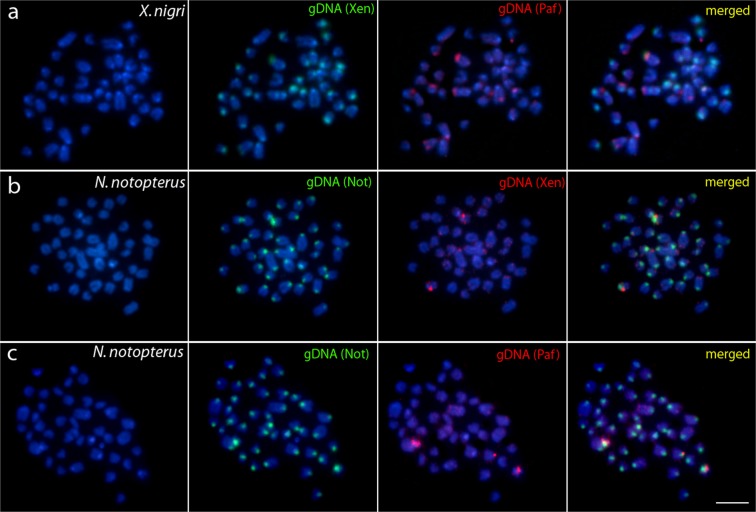
Comparative genomic hybridization (CGH) using the gDNA of *P. afer* against chromosomal background of *X. nigri* (**a**) and using the gDNA of *X. nigri* (**b**) and *P. afer* (**c**) against chromosomal background of *N. notopterus*. First column: DAPI images (blue); Second column: hybridization pattern with *X. nigri* (Xen) and *N. notopterus* (Not) gDNA probe; Third column: hybridization patterns with *X. nigri* (Xen) gDNA and *P. afer* (Paf) gDNA. Fourth column: merged images of both genomic probes and DAPI staining. The common genomic regions are depicted in yellow. Bar = 5 μm.

In the set of interspecific CGH experiments, the comparative hybridization of the probes prepared from the whole genomic DNAs (gDNAs) produced only a limited number of overlapping signals (Figs 2–4**)**. More specifically, while the gDNA probes hybridizing back against their own chromosome complements highlighted many heterochromatic blocks abundantly present in the centromeric and terminal chromosomal regions, the probes derived from the gDNA of species of other genera usually produced only weak hybridization patterns, with few consistent signals accumulated in the terminal portions of some chromosomes. Some of these signals were considerably stronger and could be related to major rDNA sites. On the other hand, when performing the intrageneric experiments in *Chitala* species, chromosomes were almost equally stained with both genomic probes (again with a stronger binding preferentially in terminal or pericentromeric heterochromatic regions), suggesting significant sequence homology. Additionally, several exclusive genome-specific signals were also detected **(**Figs 2–4**)**.

## Discussion

It is well known that orthologous genome regions from the scale of DNA sequences to entire chromosomes are sentenced to diverge over time as a byproduct of independent evolutionary histories and accompanying molecular mechanisms acting on genome. In this manner, groups with known temporal divergence offer excellent perspectives for tracking the pathways of chromosome diversification over defined evolutionary periods.

Sets of phylogenetically shared chromosomal features can indicate similar levels and patterns of chromosomal evolution in a clade. Indeed, peculiar orthologous chromosome regions can favor similar and nonrandom rearrangements acting in small or large scale within a given taxonomic group^33,34^. Among vertebrates, fishes provide very attractive models for investigation of karyotype and genome evolution in lineages with very recent divergence^35^, or that experienced separated evolutionary pathways over a long divergence time^9,12^.

According to molecular data, African and Asian Notopteridae lineages diverged during the Cretaceous period ~120 Mya^17^. Still, during the Cretaceous period, more specifically close to its end, the divergence between the African lineages (*Papyrocranus* and *Xenomystus*) is hypothesized to occur and, subsequently, the split of the Asian lineages (*Notopterus* and *Chitala*) is estimated to take place around 50 Mya (i.e. in the Tertiary period)^36^. Remarkably, even with respect to such divergent evolutionary times, the karyotype macrostructure of these fishes underwent low macrostructural changes, where only *P*. *afer* and *C*. *lopis* (2n = 50 and, 2n = 38, respectively) show some variation in their 2n. Except for *P. afer*, the karyotypes of all remaining species studied to date are composed exclusively of 42 acrocentric chromosomes^9^.

**Figure 5:**
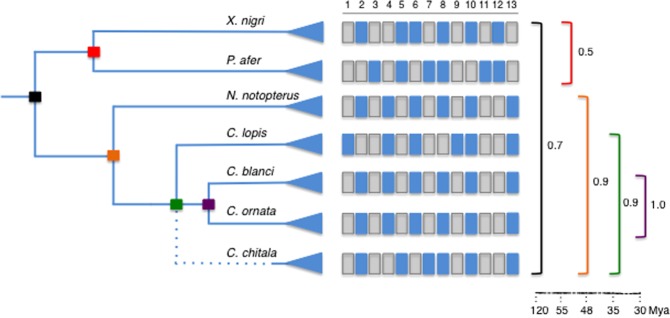
Similarity cytogenetic indexes in Notopterids clades from phylogenetic and temporal perspectives. Matrix traits: Diploid numbers - 1. 2n = 38; 2. 2n = 42; 3. 2n = 50. 18S rDNA sites: 4. unique; 5. multiple. 5S rDNA sites - 6. non synteny with 18S rDNA; 7. synteny. (TTAGGG)n sites: 8. terminal; 9. ectopic. Biarmed elements: 10. absence; 11. presence. CGH homology: 12. high genomic similarity intraclade; 13. reduced similarity.

The conservative karyotype structure and 2n among Notopteridae species are maintained over long evolutionary time evolutionary time, apparently with only slight disturbances of collinearity **(**Fig. 5**)**. This conserved cytogenetic trend contrasts with the more dynamic evolution showed by other osteoglossiform lineages. In fact, Mormyridae, a sister group of Notopteridae^17^ whose species diverged more than 100 Mya^36^ have experienced a noticeable karyotype diversification, mainly modified by pericentric inversions (NF = 52–68) (reviewed in^12,13^).

Karyotype and chromosome diversifications may accompany speciation^37–40^. During the phyletic diversification, the rate of karyotype evolution can be highly variable in phylogenetically related fish groups^41^. In fact, despite the long divergence time, several fish groups have shown no karyotype differentiation or only some minor interspecific differences. For example, in the cohort Clupeocephala, the largest clade of Teleostei with a long evolutionary history (~153 Mya), this condition is characterized by an extensive sharing of a basal karyotype with 2n = 48 acrocentric chromosomes^42,43^. Besides the macrostructural karyotype similarities, cytogenomic analyses have supported a conservative scenario regarding the genomic organization^44,45^. Although some fish groups exhibit structural karyotype diversifications, in the case of Cichlidae, for instance, chromosomal homologies have been identified by BAC-FISH, revealing large syntenic chromosome segments being maintained conserved during evolution^46^.

The high macrostructural karyotype conservation over time represents an evolutionary process defined earlier as karyotype stasis^38^ and exemplified in several fish families (e.g.^33^). Despite that, this process has not been demonstrated for all fish clades yet, and its probable causes have been delineated for some groups^47^, such as its recurrence and phylogenetic extension^34,41^. Although karyotype stasis can be associated with a recent phyletic radiation, cases where the absence of marked chromosomal changes resists to large divergence episodes suggest that the karyotype stasis can be modulated mainly by “extrinsic” and “intrinsic” causes^47^. Extrinsic causes for such low evolutionary dynamics are generally related to population structure, where: (i) the limited occurrence of biogeographic barriers^48^, (ii) range of larval pelagic phase in marine environments or active adult migration^49,50^, (iii) level of parental care, occupation of exclusive habitats and population size, can be listed^47^. On the other hand, “intrinsic” factors are usually associated with chromosome organization, leading to variable tempo of evolutionary changes in some lineages. In fact, specific chromosome characteristics related to the organization and evolutionary dynamics of particular DNA sequences can promote less stable evolutionary environment for chromosomes in a particular group of species. Among these characteristics, the amount and distribution of heterochromatin^44,45^, specific repetitive elements^51,52^, or sequences/regions that contribute to distortions during asymmetric meiotic segregation^34,41^ can be listed.

Heterochromatin is a repository of very complex sets of repetitive DNA sequences^53^, including mobile elements, notoriously involved in chromosome changes^54^, and duplications, that can propitiate substrates for illegitimate recombination, resulting in chromosome rearrangements^55–57^. Divergences in tandem repeats occur in different taxonomic levels, even in populations, and can precede the evolution of species^58^. In some characiform groups with very high karyotype diversification, the heterochromatic regions are extremely polymorphic among populations (e.g.,^59^). In contrast, reduced and homogeneous distribution of heterochromatin have been suggested as one of the probable causes of evolutionary inertia in certain chromosome sets^45^. In this sense, the high homology of orthologous heterochromatic regions in several Notopteridae species can indicate an accessory role of the repetitive elements in the karyotype divergence and it can serve as a possible explanation for the observed long-term evolutionary maintenance of karyotype macrostructure in this family.

A well-resolved phylogeny for knifefishes allowed investigations into the historical chromosome changes that occurred in this fish group. The cytogenetic divergence level was calibrated by comparative analysis of homologous chromosomal traits within and among Notopteridae clades. The prediction of growing karyotype identity is followed by all clades **(**Fig. 5**)** except for the clade formed by the African lineages *Xenomystus* and *Papyrocranus*. The cytogenetic traits analyzed indicate elevated karyotype conservatism among Asian genera/species (90%), but very low similarity between African lineages represented by *X. nigri* and *P. afer* (50%). Comparisons between African and Asian lineages (70%) are generally biased towards marked diversification observed in *P. afer*. Remarkably, this species is unique with the possession of 2n > 42, biarmed chromosomes and additionally a co-localized arrangement of 18S and 5S rDNA tandem arrays, also presented in *C. chitala*^9^, indicating a peculiar pattern of karyotype evolution.

Since the evolutionary split between African Notopteridae lineages (~55 Mya), the level of chromosome differentiation among the extant genera was lower for all Notopteridae lineages. The clade *Notopterus/Chitala* with a more recent origin (~50 Mya), presents considerably less cytogenetical divergence (90%). Molina *et al*.^41^ analyzed rates of chromosome evolution in Percomorpha groups, which comprise more than 25% of all living vertebrates, and they demonstrated considerable variation in rates of karyotype change. In this metadata study, some families showed very low rates of karyotype differentiation, reaching the patterns of chromosome stasis.

In accordance with the phylogenetic hypothesis^17,36^ the cytogenetic similarity between all *Chitala* species (diverged ~35 Mya) was lower than between *C. blanci* and *C. ornata*, which are phylogenetically closer and demonstrated identical cytogenetic characteristics. Surprisingly, the species divergence lasting approximately 30 Mya was not sufficient for fixation of any distinct cytogenetic traits, indicating a very slow temporal divergence, like other cases of karyotype stasis in fishes^47^. In *Chitala* species, the extensively shared cytogenetic features are supported by CGH experiments as these compared taxa only slightly differed in overall hybridization patterns, thus pointing to a high degree of sequence homology **(**Figs 2 and 3**)**. This condition is coincident with other vertebrate groups, whose evolutionary stasis is perceived across widely phylogenetically distributed clades by whole chromosomes that have remained mostly intact during 100 million years, like birds^51,60–62^, lizards^63^ and mammals^64,65^ species. In fishes, evidence of the high level of cytogenetic conservatism has been described among closely-related cichlid species^66^.

The set of our CGH experiments aimed to compare genomes among the different notopterid genera (*Xenomystus*, *Papyrocranus,Notopterus*, and *Chitala*), suggest an advanced stage of sequence divergence, except fot the bright signals, highly likely corresponding to NOR sites (as might be compared with previous rDNA FISH analysis^9^). Such result is typical for distantly related or substantially diverged genomes, see^67,68^). The decrease of shared sequences is hardly surprising considering the ancient time of divergence between the clades. The evolutionary genetic differentiation among Notopteridae genera is also perceived in mitochondrial and nuclear genomes using mtDNA markers and new generation sequencing technology by DArTseq markers^9,17^. The progressive temporal reduction of chromosome homology in Notopterids seems to occur slowly and suggests the cumulative action of several factors associated with chromosome evolution, such as intrachromosomal rearrangements. The internal reorganization in chromosomes is likely to be related with less identified rearrangements in fish karyotypes, such as paracentric inversions or bursts of amplification of repetitive sequences, mutations, with marked role in the karyotype evolution of several animal groups (e.g.,^69^).

The similarities between the karyotype structures in Notopteridae species raise the question: are the chromosome pairs homeologous among these species? This question is pertinent to confirm chromosome markers in groups with much diversified karyotype evolution^70^, or to establish the conservation level of syntenic groups in those with much conserved karyotype macrostructure^21^. Thus, inter-specific cross-hybridization experiments using the painting probe XNI-1 derived from the first chromosome pair of *X. nigri* karyotype were performed to help to clarify this question. In *X. nigri*, the first pair was painted, with conspicuous bright signals detected in both terminal regions (heterochromatin sites) of the chromosome after FISH. Besides, bright signals were also identified in few additional specific regions of other chromosomes of the complement. The XN-1 probe further showed diffuse and limited homology in inter-generic cross-hybridization, showing a temporal reduction of syntenic sequences. In fact, among the notopterid species of distinct genera it highlights a homeologous region, possibly heterochromatic^9^, in the centromeric region of a medium-sized acrocentric pair for all the six species. Given the larger temporal divergence among Notopteridae lineages, these shared hybridization signals are indicative of an ancestrally conserved synteny. The presence of these preserved syntenic blocks in these chromosomes open perspectives that several other homologous syntenic regions have remained conserved during the course of genome differentiation of the examined species despite the spatio-temporal isolation.

Considering birds, a group with recognized diversity, comparative mapping of orthologous genes in the Z chromosome of species belonging to different orders, confirmed the maintenance of this syntenic group. However, the linear gene order has been changed, indicating that intra-chromosomal rearrangements (mainly pericentric and paracentric inversions) occurred several times during avian evolution^71,72^.

In summary, the maintenance of the gross structure of karyotypes among the Notopteridae lineages shows that similar evolutionary processes occurred within these lineages. The conserved pattern in the clades is only disrupted by divergent numerical and structural chromosome rearrangements occurred in *P. afer*. The extant representatives from *Xenomystus, Notopterus*, and *Chitala* genera preserved a considerable level of cytogenetic identity. In general, the temporal decrease of homology among their chromosomes suggests the involvement of intrachromosomal rearrangements that likely operate to gradually reduce the degree of collinearity and conserved synteny. The tempo of chromosome evolution in this family, except by an episodic divergence in African lineages, appears to be constant over time. To sum up, our novel cytogenetic data on sub-chromosomal level corroborate the high extent of karyotype stasis in the Notopteridae family – an important teleost group for tracking the chromosome evolution, worthy further investigation with finer-scale genomic methods.

## Methods

### Mitotic chromosome preparations

**Figure 6:**
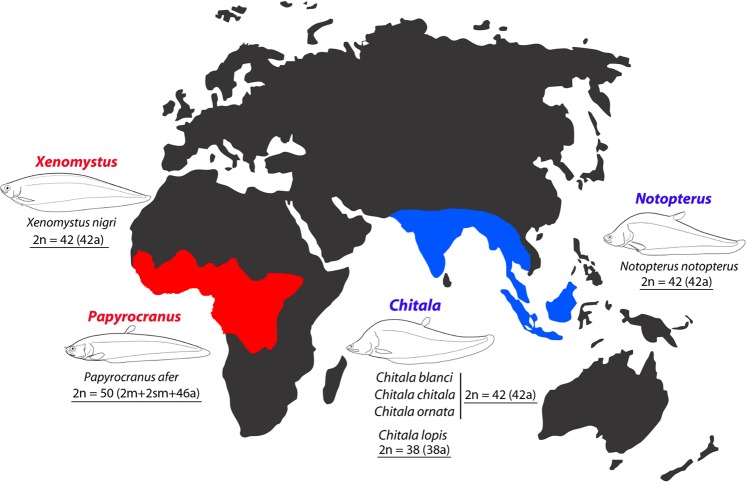
Map showing the area of distribution and the chromosomal characteristics of the seven Notopteridae species examined in this study. The geographic distribution of the living species of Notopteridae is shown in red (Africa) and blue (Asia).

Seven Notopteridae species were collected in different African and Southeast Asian River basins, as indicated in Fig. 6 and Table 1. The fishes were captured with cast-nets, placed in sealed plastic bags containing oxygen and clean water, and transported to the laboratory. The specimens were deposited in the fish collection of the Museu de Zoologia da USP, Brazil (MZUSP, vouchers 20557, 20558 and 119845). The experiments followed ethical and anesthesia conducts and were approved by the Ethics Committee on Animal Experimentation of the Universidade Federal de São Carlos (Process number CEUA 1926260315). Mitotic metaphases were prepared directly from the anterior portion of the kidney after *in vivo* colchicine treatment of the specimens following the protocol described in^73^.

**Table 1:** Collection sites of the Notopteridae species and number of individuals analyzed in this study.

Species	Sampling Site	N
*Chitala blanci Chitala chitala*	Song Khram basin, Thailand Ganges river, India	(04 ♀; 04 ♂) (05♀; 04 ♂)
*Chitala lopis*	Song Khram basin, Thailand	(12 ♀; 06 ♂)
*Chitala ornata*	Chi and Mekong basins, Thailand	(09 ♀; 07 ♂)
*Notopterus notopterus*	Chi and Mekong basins, Thailand	(06 ♀; 04 ♂)
*Papyrocranus afer*	Oluwa River, Nigeria	(19 ♀; 21 ♂)
*Xenomystus nigri*	Oluwa River, Nigeria	(13 ♀; 24 ♂)

### Chromosome microdissection, probe preparation and fluorescence *in situ* hybridization (FISH) used for WCP

For cross-species painting, we selected the first chromosome pair from the *X. nigri* complement, as it is unambiguously the largest element in the karyotype, allowing us to identify precisely both homologues after Giemsa staining. Twenty copies of this chromosome were isolated by glass-needle based microdissection, and amplified using the procedure described in Yang *et al*.^74^. The whole chromosome-derived probe (hereafter designated as XNI-1) was labelled with Spectrum-Orange-dUTP (Vysis, Downers Grove, IL, USA) through 30 cycles of secondary DOP PCR, using 1 μl of the primary amplification product^74^. The final probe cocktail was composed of 100 ng/μg of the XNI-1 probe and 60 µg of C_0_t-1 DNA (i.e. fraction of genomic DNA enriched for highly and moderately repetitive sequences) isolated from the *X. nigri* male total genomic DNA (for details, see^75^), in order to outcompete the hybridization of highly-repeated DNA sequences.

Hybridization procedure followed the protocol described in^21^ and was performed for 6 days (144 h) at 37 °C in a moist chamber. After washing procedures, the chromosomes were counterstained with DAPI (1.2 µg/ml) and mounted in antifade solution (Vector, Burlingame, CA, USA).

### Preparation of probes for CGH

**Figure 7:**
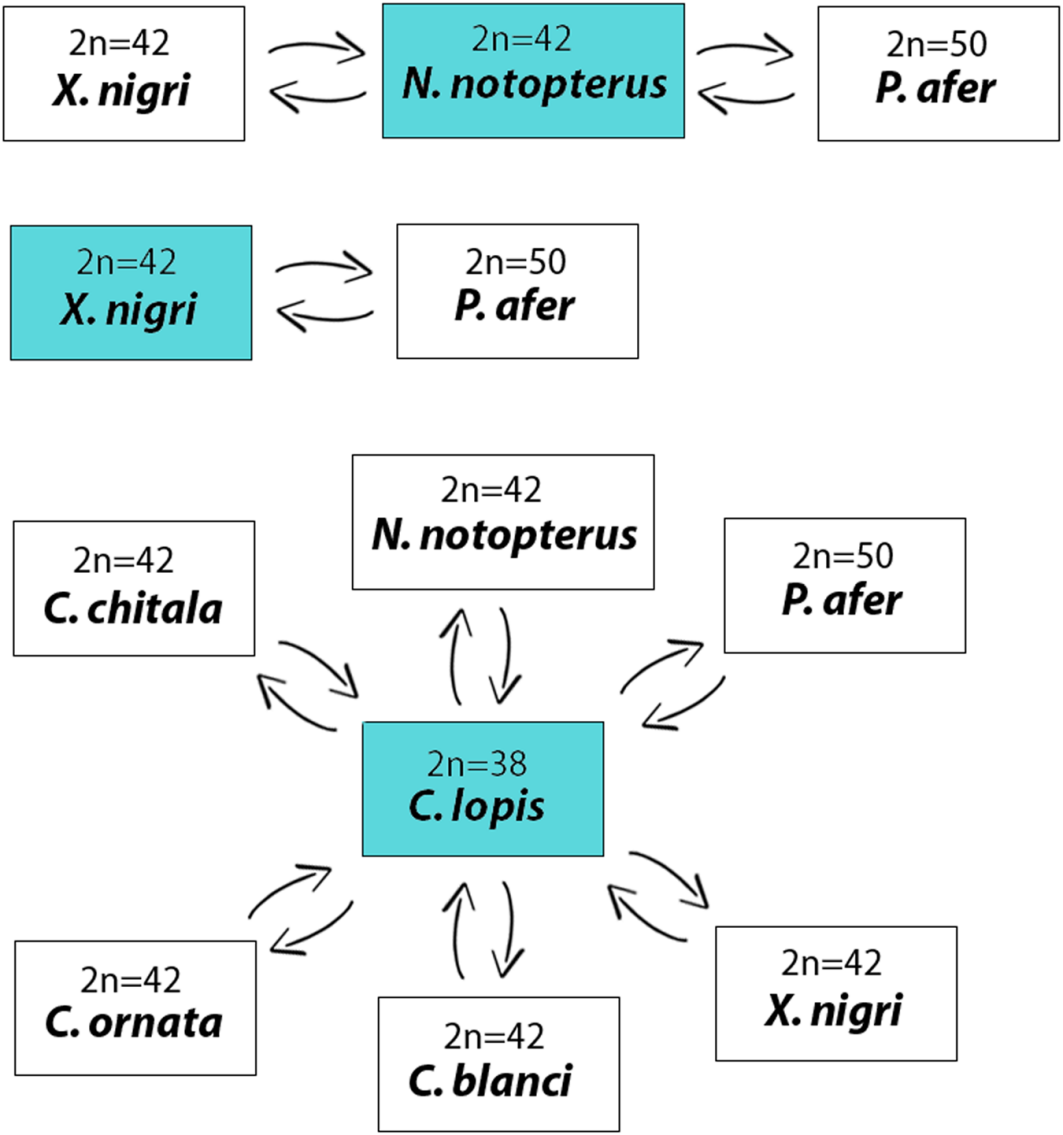
The experimental design in this study. Three different experimental designs were used in this study. In the first one, gDNA of *P. afer* and *X. nigri* were used for hybridization against chromosomal background of *N. notopterus*. In the second set of experiments, gDNA of *X. nigri* was used for hybridization against chromosomal background of *P. afer* and in the third set of experiments, gDNA probes of all Notopteridae species were used for hybridization against chromosomal background of *C. lopis*.

The genomic DNAs from male and female specimens of all species listed in Table 1 were extracted from liver tissues by the standard phenol-chloroform method^76^. Three different experimental designs were used for this study, as outlined in Fig. 7. In the first set of experiments, the gDNA of *X. nigri* and *P. afer* were compared with the gDNA of *N. notopterus* against metaphase chromosomes of the *N. notopterus*. In the second set of experiments, the gDNA of *X. nigri* was compared with the gDNA of *P. afer* against metaphase chromosomes of the *X. nigri*. And in the third set of experiments, the gDNA of all species were compared with the gDNA of *C. lopis*, in separated CGH experiments, against metaphase chromosomes of the *C. lopis*. For these purposes, gDNAs of all species were labelled either with digoxigenin-11-dUTP using DIG-nick-translation Mix (Roche, Mannheim, Germany) or biotin-16-dUTP using BIO-nick-translation Mix (Roche). For blocking the repetitive sequences in all experiments, we used C_0_t-1 DNA prepared according to Zwick *et al*.^75^. The chosen ratio of probe vs. C_0_t-1 DNA amount was based on the experiments performed in our previous studies in fishes^23,24^. We have also performed the same set of experiments without Cot1-DNA and, although we have obtained the same results, the background was higher (data not shown). Hence, the final probe cocktail for each slide was composed by 500 ng of gDNA of one species + 500 ng of gDNA corresponding to one of the comparative species + 15 μg of derived C_0_t-1 DNA of each species. The probe were ethanol-precipitated and the dry pellets were suspended in hybridization buffer containing 50% formamide, 2 × SSC, 10% SDS, 10% dextran sulfate and Denhardt’s buffer, pH 7.0.

### FISH used for CGH

For the CGH experiments, we used the methodology described in^77^, with several modifications. Briefly, a thermal aging of slides was performed prior to hybridization, at 37 °C for 2 h. Next, a treatment with RNase A (100 µg/ml, 90 min, 37 °C) took place, followed by the pepsin digestion (50 µg/ml in 10 mM HCl, 3 min, 37 °C). Denaturation of chromosomes was done in 75% formamide/2 × SSC at 74 °C for 3 min, and slides were then immediately dehydrated in 70% (cold), 85%, and 100% (RT) ethanol. The probe cocktail was denatured at 86 °C for 6 min, chilled on ice for 10 min and then applied to each slide. The hybridization was performed at 37 °C for 3 days in a dark humid chamber. Subsequently, non-specific hybridization was removed by stringent washing: once or twice in 50% formamide/2 × SSC (44 °C, 10 min each) and three times in 1 × SSC (44 °C, 7 min each). To block non-specific binding sites for antibodies, slides were incubated with 3% non-fat dried milk (NFDM) at 37 °C and subsequently hybridization signals were detected using Anti-Digoxigenin-Rhodamin (Roche) and Avidin-FITC (Sigma). Finally, the preparations were mounted in antifade containing 1.5 µg/ml DAPI (Vector).

### Microscopic analysis and image processing

At least 30 metaphase spreads per individual were analyzed to confirm the 2n, karyotype structure and FISH results. Images were captured using an Olympus BX50 microscope (Olympus Corporation, Ishikawa, Japan) with CoolSNAP and the images processed using Image Pro Plus 4.1 software (Media Cybernetics, Silver Spring, MD, USA). Chromosomes were classified as metacentric (m), subtelocentric (st) or acrocentric (a), according to their arm ratios^78^.

### Estimating levels of cytogenetic similarity among Notopteridae clades

The estimates of cytogenetic evolutionary diversification among the Notopteridae species was obtained from a data matrix with 13 specific chromosome features, including macrostructural traits (2n; presence of biarmed elements), organization of repetitive sequences in the chromosomes [e.g., rDNAs sites; (TTAGGG)n sites], and CGH homology (intraclade genomic similarity). The groups/species relationships (Notopteridae; African Notopteridae species; African/Asian Notopteridae species; Asian Notopteridae species) followed phylogenetic hypothesis based on DNA molecular markers available for family^17,36^. Cytogenetic similarity indexes were calculated for the two Notopteridae clades.

## Data Availability

All data generated or analyzed during this study are included in this published article.

## References

[1] Bănărescu, P. *Zoogeography of Fresh Waters. General Distribution and Dispersal of Freshwater**Animals* (Aula-Verlag, 1990).

[2] Greenwood, P. H., Wilson, M. V., Paxton, J. R. & Eschmeyer, W. N. *Encyclopedia of Fishes* (Academic Press: San Diego, 1998).

[3] Hilton EJ (2003). Comparative osteology and phylogenetic systematics of fossil and living bony-tongue fishes (Actinopterygii, Teleostei, Osteoglossomorpha). Zool. J. Linn. Soc..

[4] Sallan LC (2014). Major issues in the origins of ray‐finned fish (Actinopterygii) biodiversity. Biol. Rev..

[5] Darlington, P. J. *Zoogeography* (John Wiley: New York, 1957).

[6] Hilton, E. J. & Lavoué, S. A review of the systematic biology of fossil and living bony-tongue fishes, Osteoglossomorpha (Actinopterygii: Teleostei). *Neotrop. Ichthyol.* **16**, e180031 (2018).

[7] Nelson, J. S., Grande, T. C. & Wilson, M. V. H. *Fishes of the World* (John Wiley & Sons, Hoboken, 2016).

[8] Austin CM, Tan MH, Croft LJ, Hammer MP, Gan HM (2015). Whole genome sequencing of the Asian arowana (*Scleropages formosus*) provides insights into the evolution of ray-finned fishes. Genome Biol. Evol..

[9] Barby, F. *et al*. From chromosomes to genome: insights into the evolutionary relationships and biogeography of Old World knifefishes (Notopteridae; Osteoglossiformes). *Genes*, 10.3390/genes9060306 (2018). 10.3390/genes9060306 PMC6027293 29921830

[10] Bian C (2016). The Asian arowana (*Scleropages formosus*) genome provides new insights into the evolution of an early lineage of teleosts. Sci. Rep..

[11] Canitz J, Kirschbaum F, Tiedemann R (2016). Karyotype description of the African weakly electric fish *Campylomormyrus compressirostris* in the context of chromosome evolution in Osteoglossiformes. J. Physiol. Paris.

[12] Ozouf-Costaz C (2015). First insights into karyotype evolution within the family Mormyridae. Cybium.

[13] Ráb P (2016). Karyotype and mapping of repetitive DNAs in the african butterfly fish *Pantodon buchholzi*, the sole species of the family Pantodontidae. Cytogenet. Genome Res..

[14] Eschmeyer, W. N. & Fong, J. Species by family/subfamily in the Catalog of fishes, electronic version (07 November 2018). San Francisco (California Academy of Sciences) (2018).

[15] Roberts TR (1992). Systematic revision of the old world freshwater fish family Notopteridae. Ichthyol. Explor. Freshw..

[16] Vidthayanon, C. *Thailand red data: fishes* (Office of Natural Resources and Environmental Policy and Planning Bangkok, Thailand, 2005).

[17] Inoue JG, Kumazawa Y, Miya M, Nishida M (2009). The historical biogeography of the freshwater knifefishes using mitogenomic approaches: a Mesozoic origin of the Asian notopterids (Actinopterygii: Osteoglossomorpha). Mol. Phylogenet. Evol..

[18] Ráb, P., Rábová, M., Pereira, C. S., Collares-Pereira, M. J. & Pelikánová, S. Chromosome studies of European cyprinid fishes: interspecific homology of leuciscine cytotaxonomic marker–the largest subtelocentric chromosome pair as revealed by cross-species painting. *Chromosome Res* **16**, (2008). 10.1007/s10577-008-1245-3 18709543

[19] Nagamachi, C. Y. *et al*. Multiple rearrangements in cryptic species of electric knifefish, *Gymnotus carapo* (Gymnotidae, Gymnotiformes) revealed by chromosome painting. *BMC Genet*. **11**, 28 (2010). 10.1186/1471-2156-11-28 PMC2873553 20420709

[20] Symonová, R. *et al*. Genome differentiation in a species pair of coregonine fishes: an extremely rapid speciation driven by stress - activated retrotransposons mediating extensive ribosomal DNA multiplications. *BMC Evol Biol*. **13**, (2013). 10.1186/1471-2148-13-42 PMC3585787 23410024

[21] Yano CF (2017). Highly conserved Z and molecularly diverged W chromosomes in the fish genus *Triportheus* (Characiformes, Triportheidae). Heredity (Edinb)..

[22] de Freitas NL (2018). Early Stages of XY sex chromosomes differentiation in the fish *Hoplias malabaricus* (Characiformes, Erythrinidae) revealed by DNA repeats accumulation. Curr. Genomics.

[23] de Moraes RLR (2017). Evolutionary relationships and cytotaxonomy considerations in the genus *Pyrrhulina* (Characiformes, Lebiasinidae). Zebrafish.

[24] Sember A (2018). Sex chromosome evolution and genomic divergence in the fish *Hoplias malabaricus* (Characiformes, Erythrinidae). Front. Genet..

[25] Traut W, Winking H (2001). Meiotic chromosomes and stages of sex chromosome evolution in fish: zebrafish, platyfish and guppy. Chromosome Res..

[26] Schemberger MO (2011). Differentiation of repetitive DNA sites and sex chromosome systems reveal closely related group in Parodontidae (Actinopterygii: Characiformes). Genetica.

[27] Vicari MR (2011). New insights on the origin of B chromosomes in *Astyanax scabripinnis* obtained by chromosome painting and FISH. Genetica.

[28] Scudeler PES, Diniz D, Wasko AP, Oliveira C, Foresti F (2015). Whole chromosome painting of B chromosomes of the red-eye tetra *Moenkhausia sanctaefilomenae* (Teleostei, Characidae). Comp. Cytogenet..

[29] Utsunomia R (2016). Uncovering the ancestry of B chromosomes in *Moenkhausia sanctaefilomenae* (Teleostei, Characidae). PLoS One.

[30] Knytl M, Kalous L, Symonová R, Rylková K, Ráb P (2013). Chromosome studies of European cyprinid fishes: cross-species painting reveals natural allotetraploid origin of a *Carassius* female with 206 chromosomes. Cytogenet. Genome Res..

[31] Fujiwara A, Abe S, Yamaha E, Yamazaki F, Yoshida MC (1997). Uniparental chromosome elimination in the early embryogenesis of the inviable salmonid hybrids between masu salmon female and rainbow trout male. Chromosoma.

[32] Sakai C (2007). Chromosome elimination in the interspecific hybrid medaka between *Oryzias latipes* and *O. hubbsi*. Chromosome Res..

[33] Poltronieri J (2014). Comparative chromosomal mapping of microsatellites in *Leporinus* species (Characiformes, Anostomidae): unequal accumulation on the W chromosomes. Cytogenet. Genome Res..

[34] Molina WF (2014). Preferential accumulation of sex and Bs chromosomes in biarmed karyotypes by meiotic drive and rates of chromosomal changes in fishes. An. Acad. Bras. Cienc..

[35] Getlekha N (2018). Contrasting evolutionary paths among indo-pacific *Pomacentrus* species promoted by extensive pericentric inversions and genome organization of repetitive sequences. Zebrafish.

[36] Lavoué S (2016). Was Gondwanan breakup the cause of the intercontinental distribution of Osteoglossiformes? A time-calibrated phylogenetic test combining molecular, morphological, and paleontological evidence. Mol. Phylogenet. Evol..

[37] White, M. J. D. *Modes of speciatio*n. (San Francisco: WH Freeman 455p.-Illus., maps, chrom. nos General (KR, 197800185), 1978).

[38] King, M. *Species evolution: the role of chromosome change* (Cambridge University Press, 1995).

[39] Coyne, J. A. & Orr, H. A. *Speciation*. Sinauer Associates, Inc.: Sunderland, 2004).

[40] Potter S (2017). Chromosomal speciation in the genomics era: disentangling phylogenetic evolution of rock-wallabies. Front. Genet..

[41] Molina WF, Martinez PA, Bertollo LAC, Bidau CJ (2014). Evidence for meiotic drive as an explanation for karyotype changes in fishes. Mar. Genomics.

[42] Mank JE, Avise JC (2006). Phylogenetic conservation of chromosome numbers in Actinopterygiian fishes. Genetica.

[43] Brum MJ, Galetti PM (1997). Teleostei ground plan karyotype. J. Comp. Biol..

[44] Motta Neto CC, Lima-Filho PA, Araújo WC, Bertollo LAC, Molina WF (2012). Differentiated evolutionary pathways in Haemulidae (Perciformes): karyotype stasis versus morphological differentiation. Rev. Fish Biol. Fish..

[45] Neto CCM, Cioffi MB, Bertollo LAC, Molina WF (2011). Extensive chromosomal homologies and evidence of karyotypic stasis in Atlantic grunts of the genus *Haemulon* (Perciformes). J. Exp. Mar. Bio. Ecol..

[46] Mazzuchelli J, Kocher TD, Yang F, Martins C (2012). Integrating cytogenetics and genomics in comparative evolutionary studies of cichlid fish. BMC Genomics.

[47] Molina, W. F. Chromosomal changes and stasis in marine fish groups. *Fish Cytogenet*. 69–110 (2007).

[48] Brum MJI (1996). Cytogenetic studies of Brazilian marine fish. Braz. J. Genet..

[49] Soares, R. X. *et al*. Chromosomal evolution in large pelagic oceanic apex predators, the barracudas (Sphyraenidae, Percomorpha). *Genet. Mol. Res. Genet. Mol. Res* **16**, (2017). 10.4238/gmr16029644 28437559

[50] Molina WF, Galetti PM (2004). Karyotypic changes associated to the dispersive potential on Pomacentridae (Pisces, Perciformes). J. Exp. Mar. Bio. Ecol..

[51] Ellegren H (2010). Evolutionary stasis: the stable chromosomes of birds. Trends Ecol. Evol..

[52] Farré M, Robinson TJ, Ruiz‐Herrera A (2015). An integrative breakage model of genome architecture, reshuffling and evolution. Bioessays.

[53] Costa GWWF, Cioffi MB, Bertollo LAC, Molina WF (2015). Structurally complex organization of repetitive DNAs in the genome of Cobia (*Rachycentron canadum*). Zebrafish.

[54] Cordaux R, Batzer MA (2009). The impact of retrotransposons on human genome evolution. Nat. Rev. Genet..

[55] Bailey, J. A., Baertsch, R., Kent, W. J., Haussler, D. & Eichler, E. E. Hotspots of mammalian chromosomal evolution. *Genome Biol.* **5**, (2004). 10.1186/gb-2004-5-4-r23 PMC395782 15059256

[56] Peng JC, Karpen GH (2008). Epigenetic regulation of heterochromatic DNA stability. Curr. Opin. Genet. Dev..

[57] Sotero-Caio CG, Platt RN, Suh A, Ray DA (2017). Evolution and diversity of transposable elements in vertebrate genomes. Genome Biol. Evol..

[58] Feliciello I, Chinali G, Ugarković Đ (2011). Structure and population dynamics of the major satellite DNA in the red flour beetle *Tribolium castaneum*. Genetica.

[59] Kantek DLZ (2009). Chromosomal location and distribution of As51 satellite DNA in five species of the genus *Astyanax* (Teleostei, Characidae, *Incertae sedis*). J. Fish Biol..

[60] Nanda I, Karl E, Griffin DK, Schartl M, Schmid M (2007). Chromosome repatterning in three representative parrots (Psittaciformes) inferred from comparative chromosome painting. Cytogenet. Genome Res..

[61] de Oliveira EHC (2005). Chromosome reshuffling in birds of prey: the karyotype of the world’s largest eagle (Harpy eagle, *Harpia harpyja*) compared to that of the chicken (*Gallus gallus*). Chromosoma.

[62] Tagliarini MM, O’Brien P, Ferguson-Smith MA, de Oliveira EHC (2011). Maintenance of syntenic groups between Cathartidae and *Gallus gallus* indicates symplesiomorphic karyotypes in new world vultures. Genet. Mol. Biol..

[63] Pokorná, M. *et al*. Strong conservation of the bird Z chromosome in reptilian genomes is revealed by comparative painting despite 275 million years divergence. *Chromosoma* **120**, (2011). 10.1007/s00412-011-0322-0 21725690

[64] Balmus G (2007). Cross-species chromosome painting among camel, cattle, pig and human: further insights into the putative Cetartiodactyla ancestral karyotype. Chromosome Res..

[65] Kulemzina AI (2011). Chromosome painting in Tragulidae facilitates the reconstruction of Ruminantia ancestral karyotype. Chromosome Res..

[66] Valente GT, Schneider CH, Gross MC, Feldberg E, Martins C (2009). Comparative cytogenetics of cichlid fishes through genomic *in-situ* hybridization (GISH) with emphasis on *Oreochromis niloticus*. Chromosome Res..

[67] Lim KY (2007). Sequence of events leading to near‐complete genome turnover in allopolyploid *Nicotiana* within five million years. New Phytol..

[68] Majka J, Majka M, Kwiatek M, Wiśniewska H (2017). Similarities and differences in the nuclear genome organization within Pooideae species revealed by comparative genomic *in situ* hybridization (GISH). J. Appl. Genet..

[69] Matsuoka MP, Gharrett AJ, Wilmot RL, Smoker WW (2004). Genetic linkage mapping of allozyme loci in even-and odd-year pink salmon (*Oncorhynchus gorbuscha*). J. Hered..

[70] Machado MdA (2018). Extensive karyotype reorganization in the fish *Gymnotus arapaima* (Gymnotiformes, Gymnotidae) highlighted by Zoo-FISH analysis. Front. Genet..

[71] Backström N (2006). Genetic mapping in a natural population of collared flycatchers (*Ficedula albicollis*): conserved synteny but gene order rearrangements on the avian Z chromosome. Genetics.

[72] Skinner BM, Griffin DK (2012). Intrachromosomal rearrangements in avian genome evolution: evidence for regions prone to breakpoints. Heredity..

[73] Bertollo, L. A. C., Cioffi, M. B. & Moreira-Filho, O. Direct chromosome preparation from Freshwater Teleost Fishes BT - Fish cytogenetic techniques (Chondrichthyans and Teleosts). In (eds Ozouf-Costaz, C., Pisano, E., Foresti, F. & Almeida Toledo, L. F.) (Enfield USA, 10.1201/b18534-4 (2015).

[74] Yang, F., Trifonov, V., Ng, B. L., Kosyakova, N. & Carter, N. P. Generation of paint probes by flow-sorted and microdissected chromosomes BT - Fluorescence *In Situ* Hybridization (FISH)–Application Guide. In (ed. Liehr, T.), 10.1007/978-3-540-70581-9_3 (2009).

[75] Zwick MS (1997). A rapid procedure for the isolation of C0t-1 DNA from plants. Genome.

[76] Sambrook, J. & Russell, D. W. *Molecular Cloning, A Laboratory Manual* (Cold Spring Harbor Laboratory Press, 2001).

[77] Symonová, R., Sember, A., Majtánová, Z. & Ráb, P. Characterization of fish genomes by GISH and CGH. *Fish Cytogenet. Tech. Ray-Fin Fishes Chondrichthyans. CCR Press Boca Rat*. 118–131 (2015).

[78] Levan, A., Fredga, K. & Sandberg, A. A. Suggestion for a chromosome nomenclature based on centromeric position. *Hereditas* **52**, (1964).

